# Augmentative Asenapine in a Recurrent Manic Catatonic Patient with Partial Response to Clozapine

**DOI:** 10.1155/2013/503601

**Published:** 2013-09-19

**Authors:** Massimiliano Buoli, Cristina Dobrea, Alice Caldiroli, Laura Cremaschi, A. Carlo Altamura

**Affiliations:** Department of Psychiatry, University of Milan, Fondazione IRCCS Ca' Granda Ospedale Maggiore Policlinico, 20122 Milan, Italy

## Abstract

Catatonia is a severe but treatable neuropsychiatric syndrome known since the middle of the nineteenth century. It has been considered for a long time as a subtype of schizophrenia, even though this association occurs only in 10% of cases. In contrast, it is frequently observed in bipolar patients. First-line treatment consists of benzodiazepines, while in case of resistance electroconvulsive therapy (ECT) and clozapine have shown positive results. In addition, recent studies reported the efficacy of some atypical antipsychotics. The present case shows the clinical response to augmentative asenapine in a catatonic manic patient with a partial response to clozapine.

## 1. Introduction

The term Catatonia, coined in 1874 by Karl Kahlbaum, is a severe psychomotor syndrome characterized by the presence for more than 24 hours of at least two among a list of symptoms including stupor/immobility, rigidity, excessive motor activity (purposeless), staring, posturing, and autonomic alterations. Even though catatonia has been considered for a long time a subtype of schizophrenia, this syndrome frequently occurs over other conditions including mood disorders [1, 2].

According to international guidelines, high dosages of benzodiazepines are the first-line treatment for catatonia; Electroconvulsive Therapy (ECT) is to be taken into consideration when patients do not respond to benzodiazepines or rapid resolution is necessary [3]. In recent studies, some atypical antipsychotics such as risperidone and olanzapine have been successfully used, while clozapine should be reserved to resistant cases [4, 5].

In this paper we report the case of a bipolar catatonic patient who showed response to augmentative asenapine after partial response to clozapine.

## 2. Case Presentation

R. was a 46-year-old man, with a 24-year history of bipolar disorder and manic psychotic episodes requiring hospitalization. The first catatonic episode occurred in 2001 and responded to high dosages of clozapine (600 mg/day) after 6 weeks of hospitalization. During the following ten years, the patient maintained an adequate level of functioning, attending most of daily activities despite of his limited social relationships. Given that the subjective well-being had been continuing for many years, the patient decided to stop assuming clozapine in May 2012. As a consequence, a recurrence of the previous symptoms occurred, reaching its peak in September 2012, when he was admitted to our inpatients service. At the admission, R. showed euphoric mood, purposeless excessive motor activation, stereotyped movements, delusions, and visual/auditory hallucinations that required restraint and urgent hospitalization. The physical examination and routine blood tests were normal with the exception of a thalassemia trait.

At baseline, the Bush-Francis Catatonia Rating Scale [6] was administered with a total score of 33. Young-Mania Rating Scale (MRS) score was 37 [7]. His first psychopharmacological treatment included risperidone 12 mg/day combined with clonazepam 12 mg/day; it continued for five days, and then the dose of risperidone was decreased to 7 mg/day. In those days he was considerably sedated and he was not able to speak clearly, so clonazepam was stopped.

Ten days after admission no improvement in mental state was noted; R. required continuous restraint to avoid dropping down. He continued to show psychotic symptoms and psychomotor agitation. 

Therefore, on the 14th day, risperidone was replaced with zuclopenthixol (45 mg/day) and gabapentin was introduced at a dose of 600 mg/day in addition to clonazepam 3 mg/day; after three days clonazepam was stopped and gabapentin was increased to 900 mg/day.

During the three weeks of hospitalization, the patient spent his days in bed, often restrained for self-harm risk; the speech was not spontaneous and he frequently shouted to respond to his hallucinations and presented mystic delusions. Vitamins B and glucosate were introduced as he did not feed himself adequately; moreover, in order to prevent thrombosis, anticoagulant therapy with heparin was administered until the 53th day.

In order to improve agitation, impulsivity, and frequent hallucinations presented by the patient, it was planned to administer olanzapine IM 10 mg three times a day since the 19th day in combination with valproate (1000 mg daily) and zuclopenthixol (45 mg daily). After one week olanzapine injections were substituted with tablets at the same dosage (30 mg/day).

None of these treatments were effective. After one month of hospitalization, the patient was still restrained most of the time. Auditory hallucinations and impulsive behaviour persisted, R. frequently prayed shouting and often hurt himself by falling and hitting the head suddenly with great concerns for his health: in order to rule out any cerebral damage, a Cerebral Computerized Tomography had been performed at every serious downfall of the patient. Fortunately, no brain damage was detected. The interpersonal contacts were reduced and his severe symptoms showed no improvement. The Bush-Francis Catatonia Rating Scale [6] score still remained at 28, while MRS remained at 32.

On the 32nd day, the treatment was changed: olanzapine and zuclopenthixol were stopped and clozapine was introduced in combination with valproate. Clozapine was rapidly increased from 100 to 800 mg/day with a gradual improvement of symptoms and restraint was gradually reduced.

Ten days after the introduction of clozapine, valproate was stopped for its plausible pharmacokinetic interaction with clozapine (an increase in total clozapine metabolites) [8]. A reduction of hallucinations and mood improvement were observed. The speaking was more fluid and associative links were more frequently maintained. In contrast delusions and impulsivity still remained (MRS = 20).

**Figure 1:**
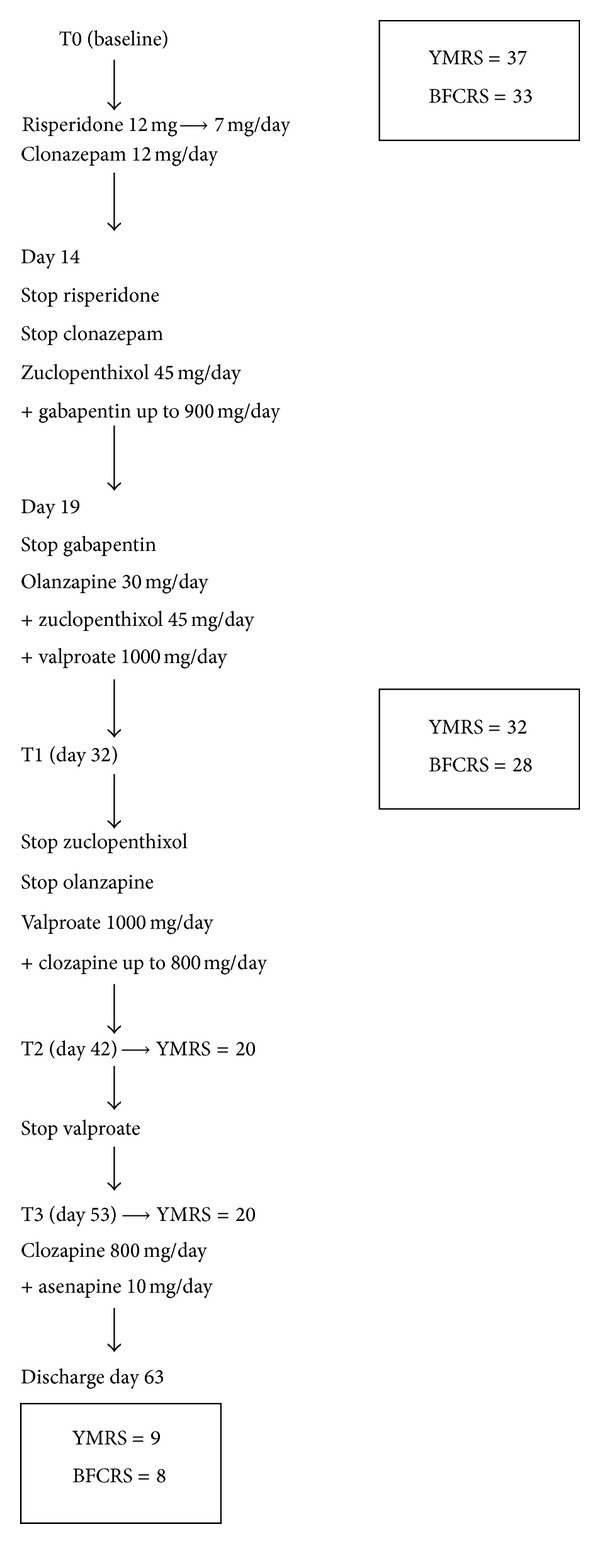
Treatment flow chart with related YMRS and BFCRS total scores.

On the 53rd day, augmentative asenapine was introduced at the dosage of 10 mg/day, with MRS scores being unchanged. The clozapine dosage was kept stable. R. showed further improvement in mood, sleep, impulsivity, psychomotor agitation, and speech. Delusion and hallucination disappeared. The patient was discharged after ten days with a Bush-Francis Catatonia Scale score of 8 and an MRS score of 9. During the subsequent 3 months, follow-up visits confirmed clinical stabilisation with clozapine 800 mg/day and asenapine 10 mg/day. The patient did not develop side effects nor during hospitalization neither during follow-up period (Figure 1).

## 3. Discussion

Catatonia is a challenging clinical condition whose etiology has not been totally clarified but it involves alteration of several neurotransmitters (GABA, glutamate, and dopamine) in different brain areas [9].

Clozapine appears efficacious in treating catatonic cases for its broad spectrum of action and low potency on D2 receptors [10]. In addition, it has been hypothesized that high doses of clozapine can modulate glutamate neurotransmission [11]. In our case clozapine improved catatonic symptoms but it resulted to be less effective in improving rapidly mania and in achieving clinical stabilization. Of note, clozapine is not actually recommended for treating manic episodes for lack of evidence [12] and this molecule can perhaps worse impulsivity and executive functioning of bipolar patients in light of its anticholinergic effects [13].

Augmentative asenapine probably was proved to be effective in improving impulsivity and clinical stabilization for absence of anticholinergic effects and its marked antagonism on D3 and 5-HT7 receptors [14]. Of note, D3 antagonism is thought to be responsible for rapid solution of manic symptoms, while 5-HT7 antagonism should prevent the switch into a major depressive episode. In light of these considerations, clozapine plus asenapine combined treatment can be thought as a treatment option in case of manic catatonia [15]. However, even though our patient did not develop side effects, an increased risk for dangerous conditions such as myocarditis or agranulocytosis has to be taken into account [16].

As a delayed efficacy of clozapine independently from asenapine augmentation has to be taken into account, further studies are needed to support this preliminary evidence about the effectiveness and tolerability of this pharmacological combination.
